# Maternal Occupational Oil Mist Exposure and Birth Defects, National Birth Defects Prevention Study, 1997–2011

**DOI:** 10.3390/ijerph16091560

**Published:** 2019-05-04

**Authors:** Miriam Siegel, Carissa M. Rocheleau, Candice Y. Johnson, Martha A. Waters, Christina C. Lawson, Tiffany Riehle-Colarusso, Jennita Reefhuis, The National Birth Defects Prevention Study

**Affiliations:** 1 Epidemic Intelligence Service, Center for Surveillance, Epidemiology and Laboratory Services, Atlanta, GA 30329, USA; 2 Division of Surveillance, Hazard Evaluations, and Field Studies, National Institute for Occupational Safety and Health, Cincinnati, OH 45226, USA; crocheleau@cdc.gov (C.M.R.); cyjohnson@cdc.gov (C.Y.J.); clawson@cdc.gov (C.C.L.); 3 Division of Applied Research and Technology, National Institute for Occupational Safety and Health, Cincinnati, OH 45226; mwaters@cdc.gov; 4 Division of Congenital and Developmental Disorders, National Center on Birth Defects and Developmental Disabilities, Atlanta, GA 30329, USA; tja4@cdc.gov (T.R.-C.); nzr5@cdc.gov (J.R.); wrm9@cdc.gov (T.N.B.D.P.S.)

**Keywords:** birth defects, congenital heart defects, oil mists, occupational exposure

## Abstract

Workers in various industries can be exposed to oil mists when oil-based fluids are aerosolized during work processes. Oil mists can be inhaled or deposited on the skin. Little research exists on the reproductive effects of oil mist exposure in pregnant workers. We aimed to investigate associations between occupational oil mist exposure in early pregnancy and a spectrum of birth defects using data from 22,011 case mothers and 8140 control mothers in the National Birth Defects Prevention Study. In total, 150 mothers were rated as exposed. Manufacturing jobs, particularly apparel manufacturing, comprised the largest groups of exposed mothers. Mothers of infants with septal heart defects (odds ratio (OR): 1.8, 95% confidence interval (CI): 1.0–3.3), and especially perimembranous ventricular septal defects (OR: 2.5, CI: 1.2–5.2), were more likely to be occupationally exposed to oil mists in early pregnancy than control mothers; and their rater-estimated cumulative exposure was more likely to be higher. This was the first U.S. study evaluating associations between oil mist exposure and a broad spectrum of birth defects. Our results are consistent with previous European studies, supporting a potential association between oil-based exposures and congenital heart defects. Further research is needed to evaluate the reproductive effects of occupational oil mist exposure.

## 1. Introduction

In 1999, the National Institute for Occupational Safety and Health (NIOSH) investigated a cluster of severe congenital heart defects (CHDs) among infants of three male employees at a steel strip manufacturing facility [1]. Two of the employees cut and packaged steel; both of their infants had hypoplastic right hearts with interrupted aortic arches. The third employee worked as a janitor and spent time in different parts of the plant, but spent the most time in the department where metals were electroplated; his infant had hypoplastic left heart syndrome. All three infants were born in 1998 in addition to 11 other infants born to plant workers that year, resulting in a prevalence of severe CHDs of 21%, compared to an expected prevalence of 0.01–0.04% for this geographic area [2].

Further investigation into births among the plant employees identified an infant with Tetralogy of Fallot born to a male plant worker in 1993. A subsequent review of insurance claims for 89 births to plant employees from 1994–2000 did not reveal any other severe CHDs, although 4 cases of atrial septal defects were identified (3 born to male employees and 1 to a female employee whom worked at the plant during pregnancy), along with 2 cases of cleft lip and palate, and 1 case of hydrocephaly (all among the offspring of male employees). This resulted in a major birth defect prevalence of 11% in the years 1994–2000. An extensive exposure assessment was conducted at the plant, but no causative agent was identified to explain the high prevalence of birth defects. While nickel was the only exposure for which measurements exceeded recommended occupational exposure levels during the investigation, workers were also exposed to copper, iron, zinc dust, a trade secret metal, and oil mists.

Oil mists arise from the use of mineral oil, a petroleum refining distillate, as either a lubricant or non-lubricant. Lubricants include metalworking fluids, engine oils, hydraulic fluids, and gear oils. Non-lubricant processes generating oil mists include agricultural oil sprays and printing ink [3]. Metalworking fluids, one of the most common sources of oil mist exposures, are used to facilitate metal cutting, inhibit rust, and lubricate and cool machinery components. They are complex mixtures that often include mineral oils, biocides, and rust inhibitors. Metalworking fluids can become aerosolized through the high shear force of moving parts, excessive heating, or spray application. The resulting oil mists can be inhaled or deposited on employees’ skin or clothing [4,5]. One potential mechanism by which occupational exposures among male employees might affect the risk of birth defects is through take-home exposure: when employees bring contaminated clothes, shoes, or work tools into their homes or automobiles where their family members (e.g., pregnant partners) can then be exposed [1]. Although not much is known about the paternal or maternal reproductive toxicity of oil mist exposure, it is possible that direct maternal occupational exposure to oil mists could have the same, or greater, effect as take-home exposure.

A majority of research on the human health effects of oil mist exposure has been related to dermal and respiratory effects and cancer [4,5,6,7]. Few studies have evaluated the relationship between oil mist exposure and birth defects. One study of paternal occupational exposures from England found no adverse association between oil mists and neural tube defects (22 exposed cases) [8]. On the other hand, occupational mineral oil exposure was found to be more prevalent among mothers of infants with CHDs (16 exposed cases) than among mothers of control infants in Finland [9], with a strong association observed with the coarctation of the aorta in a subsequent analysis (4 exposed cases) [10]. Another European study found elevated odds for occupational exposure to mineral oils in mothers of infants born with cleft palate, although the association was not statistically significant and there were only 3 exposed case mothers [11].

The current study aimed to investigate associations between estimated maternal occupational oil mist exposure during pregnancy and birth defects using population-based case-control data. Additionally, we sought to identify industries and occupations in which pregnant women were exposed to oil mists and the frequency of birth defects occurring within these industries and occupations.

## 2. Materials and Methods

### 2.1. Study Design

Data were analyzed from the National Birth Defects Prevention Study (NBDPS), a multi-center, population-based case-control study of birth defects. The NBDPS design and methods have been described elsewhere [12,13]. Briefly, the birth defects surveillance and research programs in 10 states (Arkansas, California, Georgia, Iowa, Massachusetts, New Jersey, New York, North Carolina, Texas, and Utah) conducted case ascertainment of infants and fetuses with major structural, non-chromosomal birth defects. Cases and controls were included for births on or after October 1, 1997, and with estimated dates of delivery on or before December 31, 2011. Each study site obtained institutional review board approval. Clinical geneticists reviewed each case to exclude potential syndromic cases and to classify cases as being isolated (no other major birth defects) or having multiple major birth defects [14]. All CHD cases were confirmed by echocardiography, cardiac catheterization, surgery, or autopsy. Information was reviewed by expert pediatric clinicians to classify CHD cases as simple (one single CHD or a well-defined constellation of defects recognized as one entity [15,16]), associated (common, uncomplicated combinations of CHD), or complex (three or more distinct defects). Both CHD and non-heart defects were classified as isolated (diagnosed with only one major defect) or multiple (two or more major defects occurring in different organ systems) [13]. Controls were drawn from a random sample of live births without major structural defects in the same study regions and were identified from hospital delivery logs or vital records.

Mothers completed a computer-assisted telephone interview between 6 weeks and 2 years after the estimated date of delivery. The interview included questions about various lifestyle and behavioral exposures during pregnancy, reproductive history, and occupation. This analysis included all case and control mothers who participated in NBDPS and reported working at some point during the three months before conception through the end of the pregnancy.

### 2.2. Outcomes

To be included in this analysis, we required a birth defect grouping to have at least 100 total isolated cases (simple, isolated cases for CHDs) and at least three isolated case mothers exposed to oil mists in early pregnancy. When a detailed birth defect phenotype did not meet this sample size criterion, it was grouped into a larger anatomical group if possible. Only simple isolated cases of birth defects were assessed in attempts to identify specific exposure effects for homogeneous outcome categories.

### 2.3. Exposure

Retrospective exposure assessment for oil mists was completed for maternal jobs held for one month or more during the three months before conception through the end of pregnancy for all births. A description of the exposure assessment methods has been described elsewhere [17]. In brief, each job was assigned a 1997 North American Industry Classification System (NAICS) and 2000 Standard Occupational Classification (SOC) code from information about work activities provided in the interview [18]. Two trained industrial hygienists reviewed the NAICS and SOC codes in addition to the occupational description (e.g., job title, descriptions of the company’s product/service, main job activities/duties, chemicals/substances handled, machines used on the job, and work frequency/duration) for jobs reported by mothers to assign (1) whether mothers were likely exposed to oil mists (yes/no) (including any route of exposure; i.e., inhalation, dermal, and/or ingestion) and (2) scores for estimated intensity, frequency, and rater confidence of exposure. All discrepancies were resolved by a consensus conference with a third rater. Intensity scores were quantitatively mapped to a midpoint of an intensity score category (in μg/m^3^). Cumulative exposure in the periconceptional period (defined as the month prior to conception through the first three months of pregnancy) was estimated and defined as intensity (μg/m^3^) multiplied by frequency (percentage of work hours exposed) multiplied by the typical hours per week worked at the job during this period, and the number of weeks the job was held during the periconceptional period. The resulting cumulative exposure estimate, in μg/m^3^-hours, is to be interpreted as a tool for comparison rather than a quantitative exposure measurement. If mothers held multiple jobs during the exposure window, job-specific exposure was summed across all jobs. Coders and industrial hygienists were blinded to the mothers’ case-control status.

The current analysis considered mothers exposed if at least one job held at any point during early pregnancy (i.e., one month before conception through the third month of pregnancy) was rated as having possible oil mist exposure. This period captures periods of egg maturation, fertilization and implantation, and embryo-fetal development (including organogenesis) that are considered most vulnerable to teratogens. Each exposed mother was categorized as having high exposure or low exposure based on a cutoff defined by the median estimated oil mist level among exposed control mothers.

### 2.4. Statistical Analysis

We first described the demographic characteristics among case and control mothers. Frequencies and percentages were calculated for the study site, mother’s smoking status in early pregnancy, maternal age at delivery (under 20, 20–34, and 35 years or older), mother’s body mass index (BMI) (underweight (<18.5 kg/m^2^), normal weight (18.5–<24 kg/m^2^), overweight (25–<30 kg/m^2^), and obese (>30 kg/m^2^)), maternal education (less than high school vs. high school or more), and maternal race/ethnicity (non-Hispanic white vs. non-white). P-values were calculated using chi-square tests to assess differences between cases and controls, excluding missing values.

To estimate associations between oil mist exposure in early pregnancy and individual isolated birth defects, we used logistic regression to estimate the crude odds ratios and 95% confidence intervals. Oil mist exposure was analyzed as a binary variable (i.e., any vs. none) and as a categorical variable (i.e., below and above the median cumulative exposure in controls vs. no exposure). Adjusted odds ratios and 95% confidence intervals were calculated using multivariable logistic regression controlling for the study site and smoking status a priori based on the potential variability in case and control selection across sites and the evidence for smoking as a risk factor for a spectrum of birth defects [19]. Other covariates were adjusted for in exploratory models a priori to examine potential confounding effects, but did not meet the criteria for inclusion in the final adjusted model (i.e., they were not significant in the model and/or did not produce at least a 10% change in the effect estimate of the main effect), including maternal age, BMI, education, and race/ethnicity. To further evaluate the categorical exposure variable for dose-response relationships, we calculated the *p*-trend values by treating the categorical variable as continuous in adjusted logistic regression models. A sensitivity analysis was conducted to assess how the inclusion of infants with multiple birth defects affected the estimates associated with the binary exposure variable.

A descriptive analysis was conducted to describe the most prevalent industries and occupations (I&Os) held by mothers who were occupationally exposed to oil mists in early pregnancy. Job I&Os were grouped according to NAICS and SOC codes at the 3-digit level. I&Os were included where at least 3 mothers were exposed in the early pregnancy. Jobs without enough information available to assign an industry group (i.e., NAICS “31–33”—Manufacturing) were excluded from the descriptive analysis of industry groups (*n* = 16 jobs held by 10 case and 6 control mothers that were all unexposed); all jobs had adequate information to assign an occupation group. Within each I&O group, we calculated the number of mothers who were classified as exposed to oil mists in early pregnancy and the median estimated cumulative exposure among exposed mothers. Among the exposed mothers in each I&O group, we calculated the number of total exposed cases, exposed simple isolated CHDs, and exposed isolated non-heart defects. Some mothers were included in multiple I&O groups if they held multiple jobs (*n* = 24 mothers; none were exposed to oil mists during early pregnancy in more than 1 job).

All analyses were conducted in SAS version 9.4 software (SAS Institute, Cary, NC, USA).

## 3. Results

After excluding 24 jobs due to missing or insufficiently detailed job descriptions for exposure assessment, there were 30,151 women working a total of 35,400 jobs included in this analysis. There were 22,011 case families and 8140 control families. One hundred and fifty women were rated as possibly exposed to oil mists in early pregnancy (108 case families, 42 control families) and 30,001 women were rated as unexposed (21,903 case families, 8098 control families). The median estimated exposure was 193.1 μg/m^3^-hours (range: 4.5–2160.0) in all exposed mothers. The median estimated exposure in exposed controls was 212.5 μg/m^3^-hours (range: 4.5–1071.4), leaving 78 and 72 mothers with low and high estimated exposure, respectively.

The demographic characteristics of cases and controls are shown in Table 1. Although some differences in demographic characteristics between cases and controls were statistically significant, the proportions of the study site, maternal smoking status, age at delivery, BMI, education, and race/ethnicity did not vary in a substantially meaningful way between cases and controls. Demographic characteristics of mothers exposed and unexposed to oil mists in early pregnancy are presented in the Supplementary Materials (Table S1).

Nineteen birth defect groups had a sufficient sample size for analysis (≥100 simple, isolated cases with at least 3 exposed to oil mists), including any CHD, eight specific CHD phenotypes, and 10 non-heart defects. Table 2 displays the crude and adjusted associations between estimated oil mist exposure in early pregnancy and individual birth defects. Although not statistically significant, mothers of infants born with any CHD had 1.3 times higher odds of being exposed than control mothers (95% CI: 0.8–2.0). Five out of eight specific CHD phenotypic groups (i.e., CHD groups excluding “any CHD”) displayed odds ratios above 1, although only two reached statistical significance. Crude results showed that oil mist exposure was significantly associated with all septal defects (OR: 2.0; 95% CI: 1.1–3.4), and specifically, perimembranous ventricular septal defects (VSD) (OR: 2.7; 95% CI: 1.3–5.6). There were no statistically significant associations between oil mist exposure and non-heart defects. Esophageal atresia was the only non-heart defect for which there was an odds ratio above 1 (OR: 2.4; 95% CI: 0.7–7.9). After adjusting for additional a priori covariates, estimates were similar or attenuated (Table 2). Although the main analyses were restricted to simple isolated cases to limit heterogeneity, we conducted a sensitivity analysis including both simple, isolated and multiple defects. This sensitivity analysis did not reveal any new associations; odds ratios were either very similar or slightly attenuated (Table S2).

Table 3 shows the crude and adjusted associations between estimated cumulative oil mist exposure and individual birth defects. Many comparisons could not be reported due to an inadequate number of exposed case mothers. Dose-response patterns were observed between the estimated exposure level and septal defects, particularly for perimembranous VSDs (*p*-trend = 0.02, 0.01, respectively). Mothers of infants with septal defects and specifically perimembranous VSDs were more likely to have high exposure than control mothers (OR 2.3, 95% CI: 1.1–4.7; OR 2.9, 95% CI: 1.1–7.7, respectively). No apparent dose-response patterns existed between the exposure level and non-heart defects. Results of the exposure level analyses did not change substantially when adjusting for the study site and smoking status (Table 3).

There were 10 industries and six occupations at the three-digit NAICS/SOC code level with at least three exposed case mothers (Table 4). The industries with the highest number of exposed mothers were machinery manufacturing (*n* = 27); apparel manufacturing (*n* = 24); and transportation equipment manufacturing (*n* = 18). The occupations with the highest number of exposed mothers were textile, apparel, and furnishings workers (*n* = 45); assemblers and fabricators (*n* = 35); and metal workers and plastic workers (*n* = 32). There were exposed simple isolated CHD cases in all industry and occupation groups assessed, and exposed isolated non-heart defect cases in all but one group (Table 4).

## 4. Discussion

This analysis found that mothers whose infants were born with septal heart defects, and especially perimembranous VSDs, were more likely to be exposed to oil mists during the period of one month before conception through the third month of pregnancy than control mothers; and there was some indication of a dose-response effect.

Although the small number of mothers exposed to oil mists limited statistical comparisons, the overall pattern of findings is interesting: five out of eight CHD phenotypic groups showed elevated odds ratios, while only one of 10 non-heart defects displayed an elevated odds ratio. If the results were due to chance, an approximately equal distribution of positive and inverse estimates would likely be observed for CHDs and non-heart defects. However, the observed pattern, in addition to the abovementioned significant associations, indicates a possible association between oil mist exposure and CHDs. Nine non-heart defects showed odds ratios less than 1. However, none of those estimates were statistically significant, and further dose-response analyses of non-heart defects with adequate sample size revealed estimates moved closer to the null with higher exposure, suggesting the absence of potential protective associations.

The birth defects found in association with oil mist exposure in the current analysis are similar, but not identical, to those exhibited in the birth defects cluster investigated by NIOSH in 1999 [1], namely CHDs and specifically septal heart defects. The NIOSH report found four cases of atrial septal defects during the time period of 1994–1999. The current study did not find a significant association with atrial septal defects. There were inadequate exposed sample sizes for other specific defects found in the cluster to analyze with NBDPS data, such as hypoplastic right/left heart syndrome. The observed associations between maternal oil mist exposure and CHDs are consistent with the hypothesis that birth defects among male employees working with oil mists, like those investigated by NIOSH, could occur due to take-home exposure [1]; however, we cannot rule out both a male- and female-mediated association. Exposure assessment for paternal occupational oil mist exposure was not conducted in NBDPS, in part because paternal job descriptions are only available by proxy reports made by mothers. Although proxy job descriptions often provide sufficient detail for coding for many exposures (for example, pesticides [20,21]), proxy respondents often have difficulty providing sufficient detail for unfamiliar job tasks [22] such as work in a factory (where oil mist exposure is most common in male workers).

Our findings are similar to those of studies from Finland that found maternal occupational exposure to mineral oil products [4], to be associated with CHDs, particularly the coarctation of the aorta [9,10]. Although the current sample size was too small to study coarctation of the aorta specifically, we found a statistically significant association between oil mist exposure and VSD, a defect that may occur in association with coarctation of the aorta [23]. Additionally, there was a pattern of positive associations between oil mist exposure and most CHDs analyzed. Another study found non-significantly elevated odds for occupational mineral oil exposure in mothers of infants born with oral clefts [11]. We found no association between oil mist exposure and oral clefts. However, the sample sizes in both analyses were very limited.

In the general working population, a majority of workers exposed to oil mists work in machinery, metal fabrications, and transportation equipment industries and are male [6,24]. However, maternal occupations in manufacturing have been associated with oral clefts [11,25] and anotia [18]; and maternal occupations in metalworking with amniotic band defects [18]. In the current analysis, a large portion of exposed mothers worked in textile and apparel production industries, which have a female-dominated workforce [24,26], and are not obviously associated with oil mist exposure. Workers in textile and apparel production industries can be exposed to oil mists used to lubricate machinery. Friction and heat from the machines can cause the fluids to aerosolize and be inhaled by or deposited on the skin of the operator. Studies have found maternal occupations related to textiles and apparel manufacturing to be related to birth defects such as atrioventricular septal defects [18], oral clefts [27,28], and hypospadias [29]. A 1997 NIOSH investigation of a range of symptoms reported by employees at a clothing manufacturing company found elevated rates of birth defects, stillbirths, and premature births in women who were pregnant while working at the company [30]. Most textile and apparel production workers would likely have oil mist exposure below the recommended exposure limits of 5 mg/m^3^ as a time-weighted average [31,32]. However, these limits are based on health effects in healthy adults (primarily males) and might be insufficient to protect a pregnant worker or her fetus.

Most industrial exposures are mixed, making it difficult to identify specific exposures that may be risk factors for birth defects. As an example, metalworking fluids are a mixture of chemicals which vary by use [6], including alkyl phenolic surfactants [33], solvent degreasers [34], and polycyclic aromatic hydrocarbons [4]. Alkylphenolic compounds are potential endocrine disruptors [35]. A European meta-analysis found that maternal occupational exposure to alkylphenolic compounds related to higher odds of term low birthweight [36], and another study found a positive association with hypospadias [29]. Maternal occupational exposure to trichloroethylene was found to be related to cleft palates in a European case-control study [11]. Polycyclic aromatic hydrocarbons have been shown to relate to neural tube defects [37,38,39] and gastroschisis [40]. There are also potential genotoxic properties of some metalworking fluids [41]. Information on these and other chemical and non-chemical risk factors for birth defects were either not available to include in this study or our sample size limited our ability to adjust for them.

This study is the first analysis, to the authors’ knowledge, of associations between estimated occupational exposure to oil mists and a broad spectrum of birth defects. It is also the first analysis of health effects other than cancer in an all-women sample of workers occupationally exposed to oil mists in the U.S. [42,43,44]. However, there were some limitations to the study. The analysis was limited by its small sample size due in part to the scarcity of mothers exposed to oil mists in NBDPS and the rarity of birth defects in an exposed working population. Small sample sizes yield large confidence intervals and low power. Small sample sizes also limited the ability to control for potential confounders, making residual confounding a potential explanation for the observed associations. Still, elevated association patterns were observed among several CHDs, and estimates were similar after controlling for a few covariates.

There is some potential for exposure misclassification due to the qualitative review of self-reported job characteristics and duties, especially if the details and dates reported about the jobs were insufficient or misremembered. However, the NBDPS design employed computer-assisted telephone interviews conducted by staff trained to record job histories in a standardized manner. Additionally, the timeframe for which jobs had to be recalled (i.e., before and during pregnancy) and the duration between dates of work and questionnaire administration were relatively short, likely reducing misclassification. Industrial hygiene sampling and biomarker exposure measures were not collected, meaning that it was not possible to assess the accuracy of the oil mist exposure assessment by expert raters. However, the review was conducted by an expert panel, and the inter-rater reliability for oil mist exposure assessment was high, minimizing the risk for misclassification [17]. We did not have enough detail regarding exposure to identify specific compounds within or co-occurring with oil mists that could cause reproductive toxicity. Lastly, the lack of paternal exposure information in our study could have led to (1) the potential misclassification of mothers who had working exposed partners, which could have caused the take-home exposure of mothers, and (2) an inability to consider the effects of paternal-mediated reproductive toxicity.

## 5. Conclusions

Results from the current study are consistent with previous studies finding associations between oil mist exposure and congenital heart defects, and provide additional evidence to suggest that oil mists could be a reproductive hazard. Future studies could clarify both maternally and paternally mediated mechanisms of reproductive toxicity. Further research is needed to evaluate for reproductive effects of occupational exposures to oil mists to inform occupational safety and health practices, particularly for pregnant workers, workers looking to become pregnant, and working partners of women who are pregnant or planning pregnancy.

## Figures and Tables

**Table 1:** The demographic characteristics of case mothers of infants with isolated birth defects and control mothers of infants without birth defects, NBDPS, 1997–2011.

Characteristic	Cases (*n* = 22,011)		Controls (*n* = 8140)		*p-*Value
	*n*	*%*	*n*	*%*	
Study site					<0.001
Arkansas	3021	13.7	1044	12.8	
California	2239	10.2	744	9.1	
Georgia	2524	11.5	909	11.2	
Iowa	2426	11.0	1062	13.1	
Massachusetts	3045	13.8	1084	13.3	
New Jersey	1204	5.5	407	5.0	
New York	1634	7.4	729	9.0	
North Carolina	1745	7.9	690	8.5	
Texas	1912	8.7	737	9.1	
Utah	2261	10.3	734	9.0	
Smoking status in early pregnancy					<0.001
No smoking	17,427	79.2	6602	81.1	
Any smoking	4568	20.8	1536	18.9	
Missing	16		2		
Maternal age at delivery (years)					0.04
<20	1642	7.5	565	6.9	
20–34	16,917	76.9	6366	78.2	
35+	3452	15.7	1209	14.9	
Body mass index					<0.001
Underweight	1031	4.7	372	4.6	
Normal	11,050	50.2	4298	52.8	
Overweight	5005	22.7	1837	22.6	
Obese	4372	19.9	1452	17.8	
Missing	553		181		
Maternal education					0.002
Less than high school	2456	11.2	805	9.9	
High school or more	19,513	88.8	7321	90.1	
Missing	42		14		
Maternal race/ethnicity					0.45
Non-Hispanic white	14,057	63.9	5161	63.4	
Non-white	7951	36.1	2979	36.6	
Missing	3		0		

**Table 2:** The crude and adjusted associations between any maternal occupational oil mist exposure in early pregnancy and (simple) isolated birth defects, NBDPS, 1997–2011.

Defect	Isolated Cases	Controls	Exposed Cases ^1^	Crude		Adjusted ^2^	
	*n*	*n*	*n*	OR ^3^	95% CI	OR ^3^	95% CI
Congenital heart defects (CHD)							
Any CHD	5481	8140	36	1.27	(0.82, 1.99)	1.24	(0.79, 1.95)
Conotruncal defects	1299	8140	5	0.75	(0.29, 1.89)	0.76	(0.30, 1.94)
Tetralogy of Fallot	708	8140	3	0.82	(0.25, 2.65)	0.85	(0.26, 2.75)
LVOTO defects	1068	8140	5	0.91	(0.36, 2.30)	0.95	(0.37, 2.41)
RVOTO defects	996	8140	7	1.36	(0.61, 3.05)	1.30	(0.58, 2.92)
Pulmonary valve stenosis	744	7856	6	1.55	(0.66, 3.66)	1.44	(0.61, 3.44)
Septal defects	1789	8140	18	**1.96**	**(1.13, 3.41)**	**1.84**	**(1.05, 3.25)**
Perimembranous VSD	647	8140	9	**2.72**	**(1.32, 5.61)**	**2.51**	**(1.21, 5.20)**
ASD secundum or ASD NOS	1005	8140	7	1.35	(0.61, 3.02)	1.29	(0.57, 2.94)
Non-heart defects							
Neural tube defects	1224	8140	5	0.79	(0.31, 2.00)	0.77	(0.30, 1.94)
Spina bifida	732	8140	3	0.79	(0.25, 2.57)	0.77	(0.24, 2.51)
Oral clefts	2763	8047	11	0.76	(0.39, 1.48)	0.81	(0.42, 1.58)
Cleft palate only	901	8047	3	0.64	(0.20, 2.06)	0.67	(0.21, 2.16)
Cleft lip with cleft palate	1159	8047	5	0.83	(0.33, 2.09)	0.87	(0.34, 2.22)
Cleft lip without cleft palate	703	8047	3	0.82	(0.25, 2.64)	0.89	(0.27, 2.87)
Esophageal atresia	242	8140	3	2.42	(0.74, 7.86)	2.50	(0.76, 8.18)
Hypospadias	1776	4157	7	0.82	(0.35, 1.94)	0.91	(0.38, 2.21)
Craniosynostosis	1017	8140	4	0.76	(0.27, 2.13)	0.80	(0.28, 2.23)
Gastroschisis	847	8140	3	0.69	(0.21, 2.22)	0.73	(0.22, 2.39)

^1^ Exposed controls *n* = 42 for all defects except pulmonary valve stenosis (*n* = 41) and hypospadias (*n* = 20); ^2^ Adjusted for study site and smoking status (observations with missing smoking status excluded); ^3^ Bold font indicates statistically significant associations. OR: Odds ratio; CI: Confidence interval; LVOTO: Left ventricular outflow tract obstruction; RVOTO: Right ventricular outflow tract obstruction; VSD: Ventricular septal defect; ASD: Atrial septal defect; NOS: Not otherwise specified.

**Table 3:** The crude and adjusted associations between estimated cumulative oil mist exposure level and (simple) isolated birth defects, NBDPS, 1997–2011.

Defect ^1^	Low Exposure ^2^	High Exposure ^2^	Crude				Adjusted ^3^				*p-*Trend ^2^
			Low Exposure		High Exposure		Low Exposure		High Exposure		
	*n* cases	*n* cases	OR	95% CI	OR ^4^	95% CI	OR	95% CI	OR ^4^	95% CI	
Congenital heart defects (CHD)											
Any CHD	17	19	1.26	(0.66, 2.42)	1.28	(0.70, 2.38)	1.21	(0.63, 2.32)	1.28	(0.69, 2.37)	0.35
Septal defects	7	11	1.60	(0.68, 3.79)	**2.29**	**(1.11, 4.72)**	1.37	(0.57, 3.29)	**2.34**	**(1.11, 4.91)**	**0.02**
Perimembranous VSD	4	5	2.54	(0.87, 7.45)	**2.89**	**(1.09, 7.65)**	2.17	(0.73, 6.43)	**2.84**	**(1.06, 7.59)**	**0.01**
Non-heart defects											
Oral clefts	5	6	0.73	(0.27, 1.94)	0.79	(0.32, 1.96)	0.80	(0.30, 2.14)	0.82	(0.33, 2.02)	0.56
Cleft lip with or without cleft palate	3	5	0.65	(0.19, 2.18)	0.98	(0.37, 2.60)	0.72	(0.21, 2.43)	1.02	(0.38, 2.71)	0.85

^1^ Only defects with ≥3 cases with low exposure and high exposure are presented; ^2^ *n* = 20 controls with low exposure and *n* = 22 controls with high exposure for all defects presented; ^3^ Adjusted for study site and smoking status (observations with missing smoking status excluded); ^4^ Bold font indicates statistically significant associations. OR: Odds ratio; CI: Confidence interval; VSD: Ventricular septal defect.

**Table 4:** The number of mothers occupationally exposed to oil mists in early pregnancy and cases by industry and occupation, NBDPS, 1997–2011.

3-Digit Code	Title	Workers Exposed to Oil Mists in Early Pregnancy ^1^		Median Exposure (µg/m^3^-hours)	Exposed Cases ^3^	Exposed Simple Isolated CHDs	Exposed Isolated Non-Heart Defects
		*n*	(%) ^2^		*n*	*n*	*n*
	Industry (NAICS)						
313	Textile Mills	5	(12.5)	212.6	4	NR	NR
314	Textile Product Mills	12	(23.5)	191.3	10	NR	3
315	Apparel Manufacturing	24	(19.7)	180.7	20	8	9
326	Plastics and Rubber Products Manufacturing	12	(10.8)	210.4	10	5	NR
331	Primary Metal Manufacturing	3	(11.5)	191.3	3	NR	NR
332	Fabricated Metal Product Manufacturing	15	(15.8)	287.0	12	7	4
333	Machinery Manufacturing	27	(21.1)	258.3	19	6	10
335	Electrical Equipment, Appliance, and Component Manufacturing	9	(10.1)	142.8	7	3	3
336	Transportation Equipment Manufacturing	18	(14.5)	255.8	14	NR	8
339	Miscellaneous Manufacturing	6	(3.3)	191.3	4	NR	3
	Occupation (SOC)						
511	Supervisors, Production Workers	5	(8.9)	215.2	4	NR	NR
512	Assemblers and Fabricators	35	(21.0)	191.3	25	6	13
514	Metal Workers and Plastic Workers	32	(36.8)	321.4	26	9	12
516	Textile, Apparel, and Furnishings Workers	45	(17.1)	191.3	32	10	13
519	Other Production Occupations	19	(3.5)	191.3	12	4	5
537	Material Moving Workers	5	(1.0)	191.3	3	NR	0

^1^ No exposed workers fall into more than one industry or occupation category; ^2^ The percentage of workers in an industry or occupation group that were exposed to oil mists in early pregnancy; ^3^ Total birth defect cases exposed to oil mists in early pregnancy; NR: Not reportable (*n* < 3).

## Supplementary Materials

The following are available online at https://www.mdpi.com/1660-4601/16/9/1560/s1: Table S1: Demographic characteristics of mothers occupationally exposed and unexposed to oil mists in early pregnancy, NBDPS 1997–2011, Table S2: Crude and adjusted associations between maternal occupational oil mist exposure in early pregnancy and (simple) isolated or multiple birth defects, NBDPS 1997–2011.
