# Supplementary material for: Maternal Occupational Oil Mist Exposure and Birth Defects, National Birth Defects Prevention Study, 1997–2011
Source: Int J Environ Res Public Health. 2019 May 4;16(9):1560. doi: 10.3390/ijerph16091560 (PMC6539329; doi:10.3390/ijerph16091560)
Supplement: Supplementary file 1 [file ijerph-16-01560-s001.pdf]

Table S1: Demographic characteristics of mothers occupationally exposed and unexposed to oil mists in early pregnancy, NBDPS, 1997–2011

	Exposed (n=150)		Unexposed (n=30,001)		
	n	%	*n*	%	*p-value*
Study site					<.001
Arkansas	41	27.3	4,024	13.4	
California	6	4.0	2,977	9.9	
Georgia	12	8.0	3,421	11.4	
Iowa	33	22.0	3,455	11.5	
Massachusetts	9	6.0	4,120	13.7	
New Jersey	11	7.3	1,600	5.3	
New York	8	5.3	2,355	7.9	
North Carolina	14	9.3	2,421	8.1	
Texas	8	5.3	2,641	8.8	
Utah	8	5.3	2,987	10.0	
Smoking status in early pregnancy					0.05
No smoking	110	73.3	23,919	79.8	
Any smoking	40	26.7	6,064	20.2	
Missing	0		18		
Maternal age at delivery (years)					0.66
< 20	13	8.7	2,194	7.3	
20 - 34	117	78.0	23,166	77.2	
35 +	20	13.3	4,641	15.5	
Body mass index					0.12
Underweight	5	3.3	1,398	4.7	
Normal	63	42.0	15,285	51.0	
Overweight	44	29.3	6,798	22.7	
Obese	30	20.0	5,794	19.3	
Missing	8		726		
Maternal education					<.001
Less than high school	35	23.3	3,226	10.8	
High school or more	115	76.7	26,719	89.2	
Missing	0		56		
Maternal race/ethnicity					<.001
Non-Hispanic white	75	50.0	19,143	63.8	
Non-white	75	50.0	10,855	36.2	
Missing	0		3		

Table S2: Crude and adjusted associations between any maternal occupational oil mist exposure in early pregnancy and [simple] isolated or multiple birth defects, NBDPS, 1997–2011

Defect	Isolated + multiple cases [1]	Controls	Exposed cases	Crude			Adjusted [2]		
	n	*n*	*n*	*OR*	*95% CI*		*OR*	*95% CI*	
Congenital heart defects (CHD)									
Any CHD	8647	8140	53	1.19	(0.79,	1.78)	1.20	(0.80,	1.81)
Conotruncal defects	1874	8140	9	0.93	(0.45,	1.91)	0.97	(0.47,	2.00)
Tetralogy of Fallot	890	8140	3	0.65	(0.20,	2.11)	0.68	(0.21,	2.20)
LVOTO defects	1554	8140	7	0.87	(0.39,	1.95)	0.92	(0.41,	2.06)
RVOTO defects	1483	8140	8	1.05	(0.49,	2.23)	1.01	(0.47,	2.17)
Pulmonary valve stenosis	1097	7856	6	1.05	(0.44,	2.47)	0.99	(0.42,	2.36)
Septal defects	3246	8140	26	1.56	(0.95,	2.54)	1.55	(0.94,	2.55)
Perimembranous VSD	1175	8140	15	**2.49**	**(1.38,**	**4.51)**	**2.47**	**(1.36,**	**4.49)**
ASD secundum or ASD NOS	1646	8140	12	1.42	(0.74,	2.70)	1.43	(0.74,	2.77)
Non-heart defects									
Neural tube defects	1407	8140	5	0.69	(0.27,	1.74)	0.67	(0.26,	1.69)
Spina bifida	830	8140	3	0.70	(0.22,	2.26)	0.69	(0.21,	2.22)
Oral clefts	3213	8047	15	0.89	(0.50,	1.61)	0.95	(0.53,	1.72)
Cleft palate only	1099	8047	6	1.05	(0.44,	2.47)	1.09	(0.46,	2.58)
Cleft lip with cleft palate	1358	8047	6	0.85	(0.36,	1.99)	0.91	(0.38,	2.14)
Cleft lip without cleft palate	756	8047	3	0.76	(0.23,	2.46)	0.82	(0.25,	2.66)
Esophageal atresia	562	8140	5	1.73	(0.68,	7.86)	1.82	(0.71,	4.63)
Hypospadias	1990	4157	7	0.73	(0.31,	1.73)	0.81	(0.34,	1.96)
Craniosynostosis	1128	8140	4	0.69	(0.25,	1.92)	0.72	(0.26,	2.01)
Gastroschisis	932	8140	3	0.62	(0.19,	2.01)	0.67	(0.21,	2.20)

[1] Defects that are multiple may fall into more than one defect group

[2] Adjusted for study site and smoking status (observations with missing smoking status excluded)

OR: Odds ratio; CI: Confidence interval; LVOTO: Left ventricular outflow tract obstruction; RVOTO: Right ventricular outflow tract obstruction; VSD: Ventricular septal defect; ASD: Atrial septal defect; NOS: Not otherwise specified
